# Supplementary material for: Highly structured populations of deep-sea copepods associated with hydrothermal vents across the Southwest Pacific, despite contrasting life history traits
Source: PLoS One. 2023 Nov 6;18(11):e0292525. doi: 10.1371/journal.pone.0292525 (PMC10627453; doi:10.1371/journal.pone.0292525)
Supplement: S2 File — (DOCX) [file pone.0292525.s002.docx]

**Title**

Highly structured populations of deep-sea copepods associated with hydrothermal vents across the Southwest Pacific, despite contrasting life history traits.

**Authors**

Coral Diaz-Recio Lorenzo^1,2,5^**^*^**, Tasnim Patel^3^, Eve-Julie Arsenault-Pernet^4^, Camille Poitrimol^4,5^, Didier Jollivet^5^, Pedro Martinez Arbizu^6^, Sabine Gollner^1^

**Affiliations**

^1^NIOZ Royal Netherlands Institute for Sea Research, Landsdiep 4, 1797 SZ, ‘t Horntje (Texel), The Netherlands.
^2^Utrecht University, Budapestlaan 4, 3584 CD, Utrecht, The Netherlands.

^3^ Royal Belgian Institute of Natural Sciences, Vautierstraat 29, B-1000, Brussels.

^4^ Biologie et Ecologie des Ecosystèmes marins Profonds (UMR BEEP UBO-CNRS-IFREMER), IFREMER Centre de Bretagne, F 29280, Plouzané, France

^5^Adaptation et Diversité en Milieu Marin (AD2M), Station Biologique de Roscoff, Sorbonne Université, CNRS, Roscoff, 29680, France

^6^ Senckenberg am Meer, German Center for Marine Biodiversity Research, Südstrand 44, 26382 Wilhelmshaven, Germany.

***Corresponding author**
Email: [coral.diazrecio@nioz.nl](mailto:coral.diazrecio@nioz.nl)
Address: Landsdiep 4, 1797 SZ, ‘t Horntje (Texel), The Netherlands. Department of Ocean Systems (OCS).

**Keywords:** Hydrothermal vent, meiofauna, copepods, diversity, demography, traits

# Abstract

Hydrothermal vents are extreme environments, where abundant communities of copepods with contrasting life history traits co-exist along hydrothermal gradients. Here, we discuss how these traits may contribute to the observed differences in molecular diversity and population genetic structure. Samples were collected from vent locations across the globe including active ridges and back-arc basins and compared to existing deep-sea hydrothermal vent and shallow water data, covering a total of 22 vents and 3 non-vent sites. A total of 806 sequences of mtDNA from the *Cox1* gene were used to reconstruct the phylogeny, haplotypic relationship and demography within vent endemic copepods (Dirivultidae, *Stygiopontius* spp.) and non-vent-endemic copepods (Ameiridae, Miraciidae and Laophontidae). A species complex within *Stygiopontius lauensis* was studied across five pacific back-arc basins at eight hydrothermal vent fields, with cryptic species being restricted to the basins they were sampled from. Copepod populations from the Lau, North Fiji and Woodlark basins are undergoing demographic expansion, possibly linked to an increase in hydrothermal activity in the last 10 kya. Highly structured populations of *Amphiascus* aff. *varians* 2 were also observed from the Lau to the Woodlark basins with populations also undergoing expansion. Less abundant harpacticoids exhibit little to no population structure and stable populations. This study suggests that similarities in genetic structure and demography may arise in vent-associated copepods despite having different life history traits. As structured meta-populations may be at risk of local extinction should major anthropogenic impacts, such as deep-sea mining, occur, we highlight the importance of incorporating a trait-based approach to investigate patterns of genetic connectivity and demography, particularly regarding area-based management tools and environmental management plans.

S1) Species delimitation

1. Assemble Species by Automatic Partitioning (ASAP, formerly ABGD)

**Harpacticoid species delimitation**


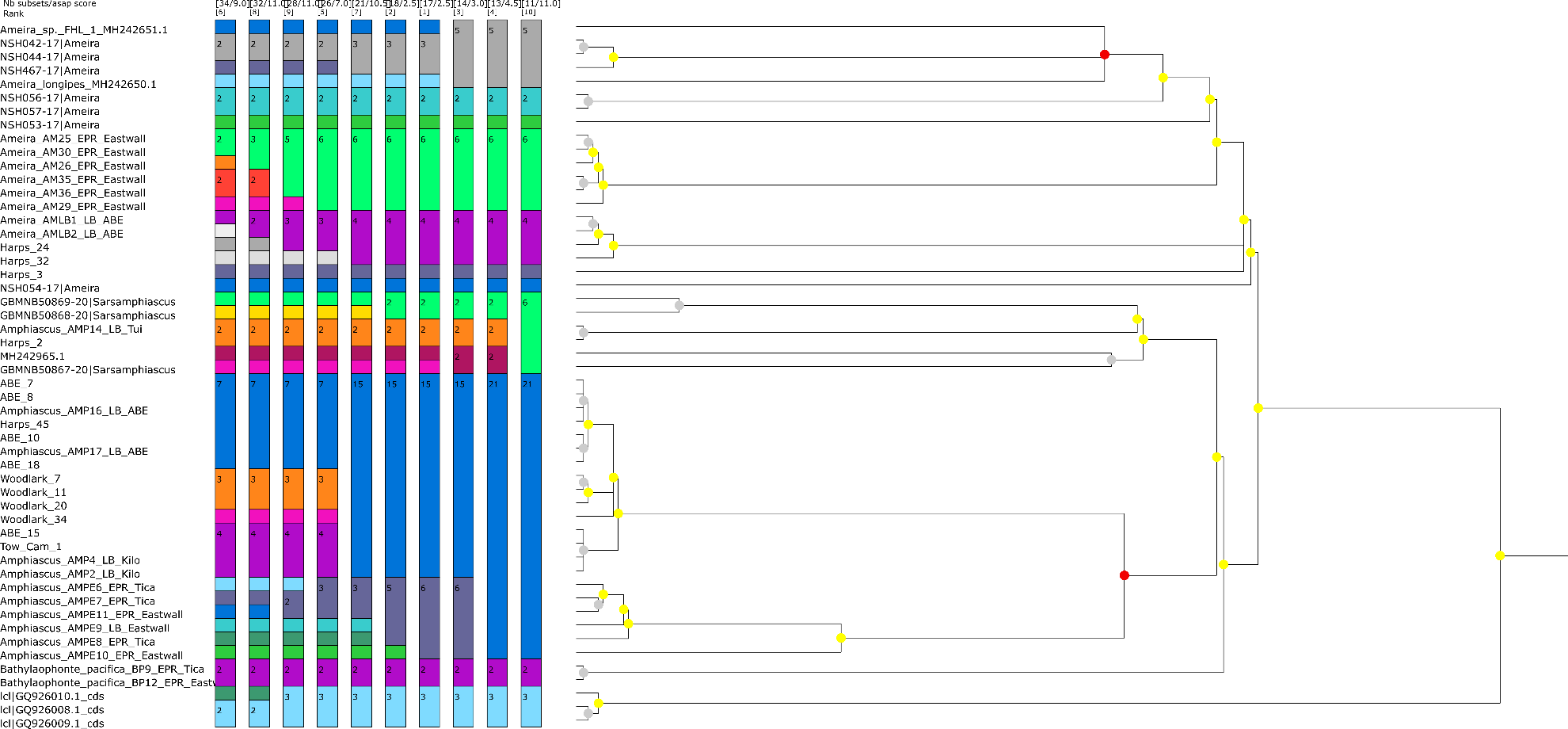


**Fig 1**: Species delimitation using Assemble Species by Automatic Partitioning (ASAP) software for the following groups of harpacticoid copepods: *Ameira*, *Amphiascus*, and *Bathylaophonte*. Lowest ASAP score is assigned to the optimal number of groups (17 species, ASAP score = 2.5). The ASAP score confirms the species hypotheses that the *Ameira* sp. 4 and 5 are two separate single species and that *Amphiascus* aff. *varians* 1 and 2 are two separate single species.

***Stygiopontius lauensis* species complex delimitation**


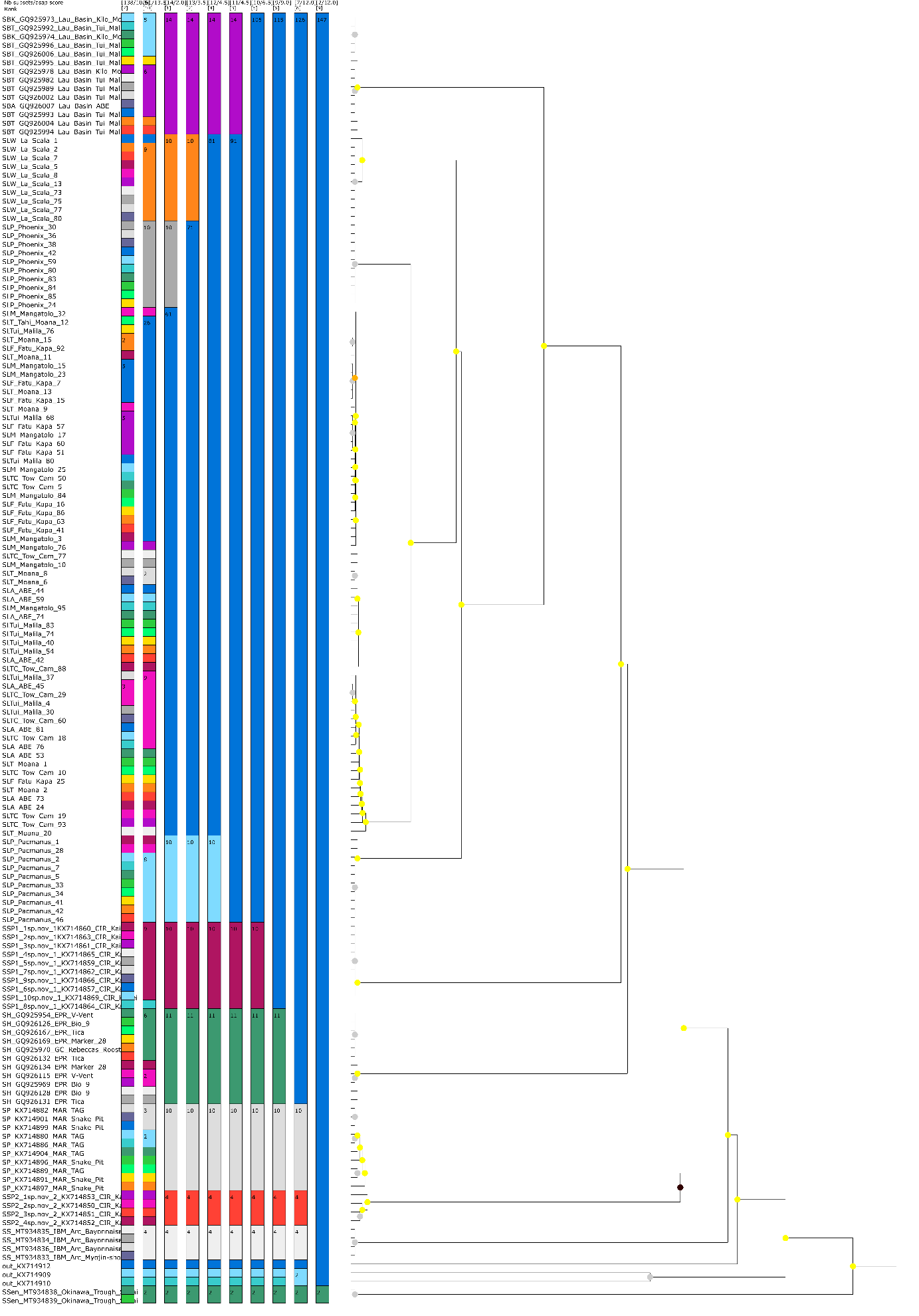


**Fig 2**: Species delimitation using Assemble Species by Automatic Partitioning (ASAP) software for the Dirivultidae. Lowest ASAP score is assigned to the optimal number of groups (17 species, asap score = 2.5). The ASAP score confirms the species hypotheses that *S. lauensis, S.* aff. *lauensis* 1, 2, and 3 are all separate, single species.

1. **Bayesian Poisson Tree Process (bPTP)**

Harpacticoida


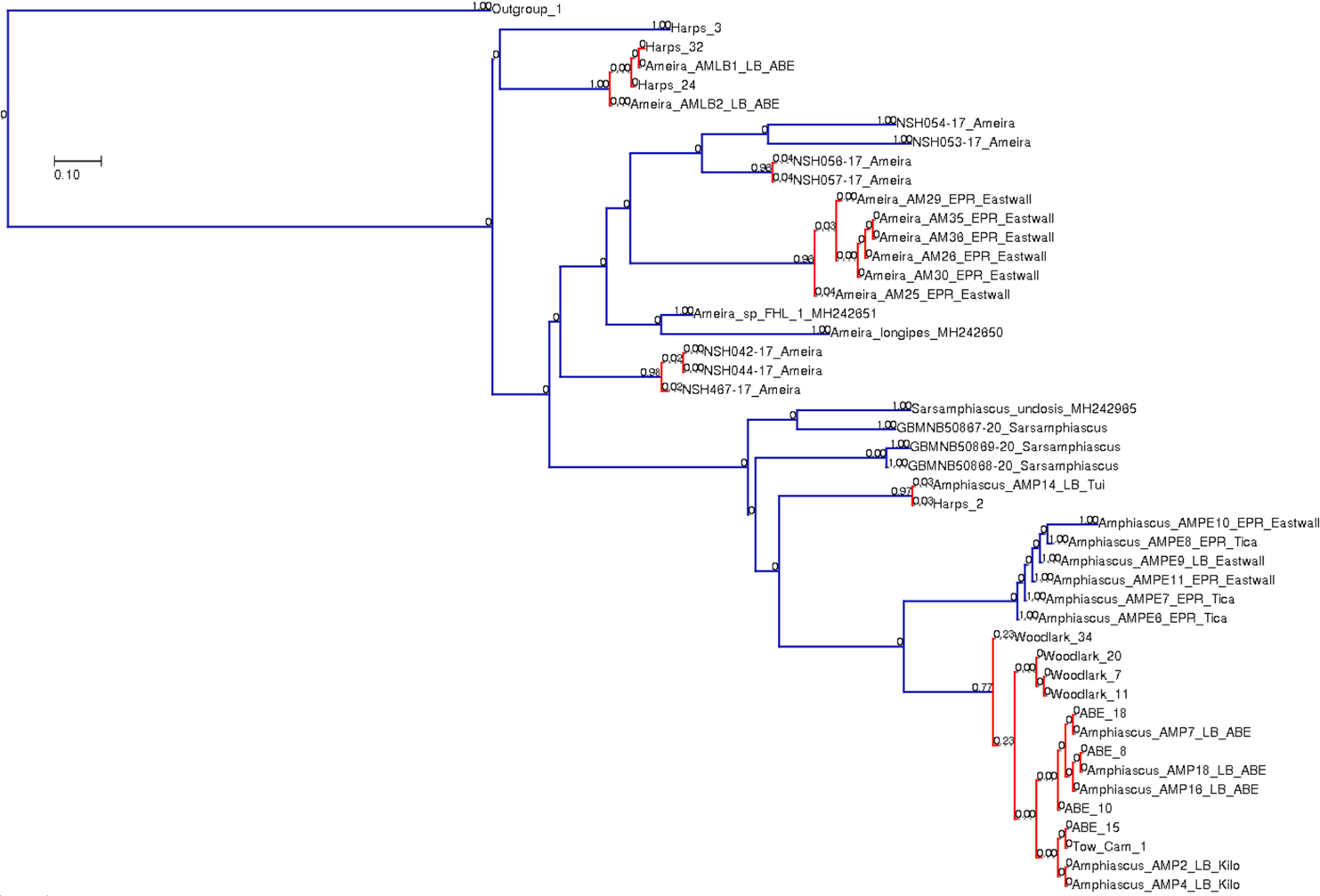


**Fig 3**: Species delimitation using Bayesian Poisson Tree Process (*bPTP*) for the harpacticoid alignment. Blue lines denote support for delimitation whereas red lines do not. *bPTP* confirms the species hypotheses that the *Ameira* sp. 4 and 5 are two separate single species and that *Amphiascus* aff. *varians* 1 and 2 are two separate single species. It also supports delimitation within the  *A.* aff. *varians*  group.

Dirivultidae


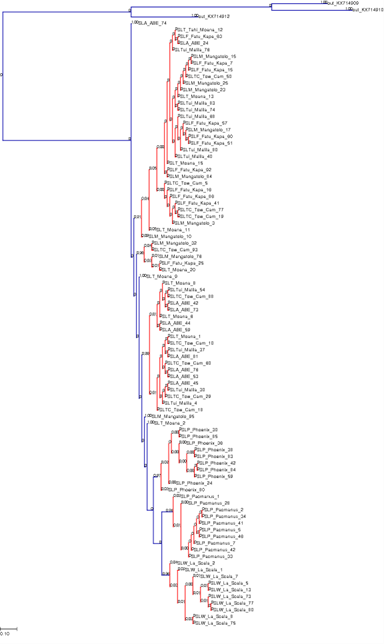


**Fig 4**: Species delimitation using Bayesian Poisson Tree Process (*bPTP*) for the dirivultid alignment. Blue lines denote support for delimitation whereas red lines do not*.* Results of *bPTP* confirm the species hypotheses that *S. lauensis, S.* aff. *lauensis* 1, 2, and 3 are all separate, single species.

1. **Bayes Factor delimitation (BFD)**

**Table 1**: Results of Bayes Factor Delimitation (BFD) for *Ameira* sp. 4 and 5, *Amphiascus* aff. *varians* 1 and 2, and the *Stygiopontius lauensis* complex. The BFD supports the single species hypothesis for both *Ameira* sp. 4 and 5 as well as for *Amphiascus* aff. *varians* 1 and 2. Support for the multispecies hypothesis is given for the *Stygiopontius lauensis* complex. The Bayes Factor is calculated using the equation in the Statistical parameters column, where support for single species is given when the difference between the marginal likelihoods is less than the twice the sum of the standard deviation and support for multiple species is given when the difference is higher.

| **Statistical parameter** | **Species hypothesis** | ***A*. aff. *varians* 1** | ***A*. aff. *varians* 2** | ***S. lauensis* complex** |
| --- | --- | --- | --- | --- |
| Marginal Likelihood | Multiple | -1585.74 | -1483.53 | -3346.69 |
|  | Single | -1563.87 | -1489.35 | -3425.61 |
| Standard deviation | Multiple | 2.04 | 2.08 | 4.49 |
|  | Single | 1.62 | 2.23 | 5.4 |
| 𝛿(ML) < 2*Σ(SD) | TRUE/FALSE | TRUE=single species | TRUE=single species | FALSE=multiple species |
