# Supplementary material for: Highly structured populations of deep-sea copepods associated with hydrothermal vents across the Southwest Pacific, despite contrasting life history traits
Source: PLoS One. 2023 Nov 6;18(11):e0292525. doi: 10.1371/journal.pone.0292525 (PMC10627453; doi:10.1371/journal.pone.0292525)
Supplement: S3 File — (DOCX) [file pone.0292525.s003.docx]

**Title**

Highly structured populations of deep-sea copepods associated with hydrothermal vents across the Southwest Pacific, despite contrasting life history traits.

**Authors**

Coral Diaz-Recio Lorenzo^1,2,5^**^*^**, Tasnim Patel^3^, Eve-Julie Arsenault-Pernet^4^, Camille Poitrimol^4,5^, Didier Jollivet^5^, Pedro Martinez Arbizu^6^, Sabine Gollner^1^

**Affiliations**

^1^NIOZ Royal Netherlands Institute for Sea Research, Landsdiep 4, 1797 SZ, ‘t Horntje (Texel), The Netherlands.
^2^Utrecht University, Budapestlaan 4, 3584 CD, Utrecht, The Netherlands.

^3^ Royal Belgian Institute of Natural Sciences, Vautierstraat 29, B-1000, Brussels.

^4^ Biologie et Ecologie des Ecosystèmes marins Profonds (UMR BEEP UBO-CNRS-IFREMER), IFREMER Centre de Bretagne, F 29280, Plouzané, France

^5^Adaptation et Diversité en Milieu Marin (AD2M), Station Biologique de Roscoff, Sorbonne Université, CNRS, Roscoff, 29680, France

^6^ Senckenberg am Meer, German Center for Marine Biodiversity Research, Südstrand 44, 26382 Wilhelmshaven, Germany.

***Corresponding author**
Email: [coral.diazrecio@nioz.nl](mailto:coral.diazrecio@nioz.nl)
Address: Landsdiep 4, 1797 SZ, ‘t Horntje (Texel), The Netherlands. Department of Ocean Systems (OCS).

**Keywords:** Hydrothermal vent, meiofauna, copepods, diversity, demography, traits

# Abstract

Hydrothermal vents are extreme environments, where abundant communities of copepods with contrasting life history traits co-exist along hydrothermal gradients. Here, we discuss how these traits may contribute to the observed differences in molecular diversity and population genetic structure. Samples were collected from vent locations across the globe including active ridges and back-arc basins and compared to existing deep-sea hydrothermal vent and shallow water data, covering a total of 22 vents and 3 non-vent sites. A total of 806 sequences of mtDNA from the *Cox1* gene were used to reconstruct the phylogeny, haplotypic relationship and demography within vent endemic copepods (Dirivultidae, *Stygiopontius* spp.) and non-vent-endemic copepods (Ameiridae, Miraciidae and Laophontidae). A species complex within *Stygiopontius lauensis* was studied across five pacific back-arc basins at eight hydrothermal vent fields, with cryptic species being restricted to the basins they were sampled from. Copepod populations from the Lau, North Fiji and Woodlark basins are undergoing demographic expansion, possibly linked to an increase in hydrothermal activity in the last 10 kya. Highly structured populations of *Amphiascus* aff. *varians* 2 were also observed from the Lau to the Woodlark basins with populations also undergoing expansion. Less abundant harpacticoids exhibit little to no population structure and stable populations. This study suggests that similarities in genetic structure and demography may arise in vent-associated copepods despite having different life history traits. As structured meta-populations may be at risk of local extinction should major anthropogenic impacts, such as deep-sea mining, occur, we highlight the importance of incorporating a trait-based approach to investigate patterns of genetic connectivity and demography, particularly regarding area-based management tools and environmental management plans.

**S2) Bayesian Posterior Probability tree parameters**


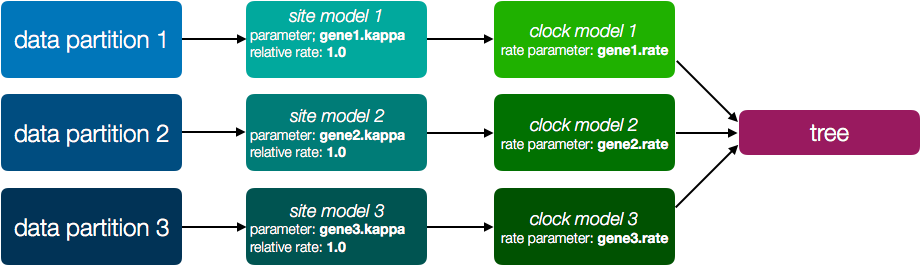


**Fig 1**: Model selection workflow schematic for BPP using BEAST. Model implies independent variation in evolutionary forces between the data partitions and the branches within the partitions but concatenates these results into a final tree. Image from BEAST tutorial (<https://beast.community/constructing_models>).


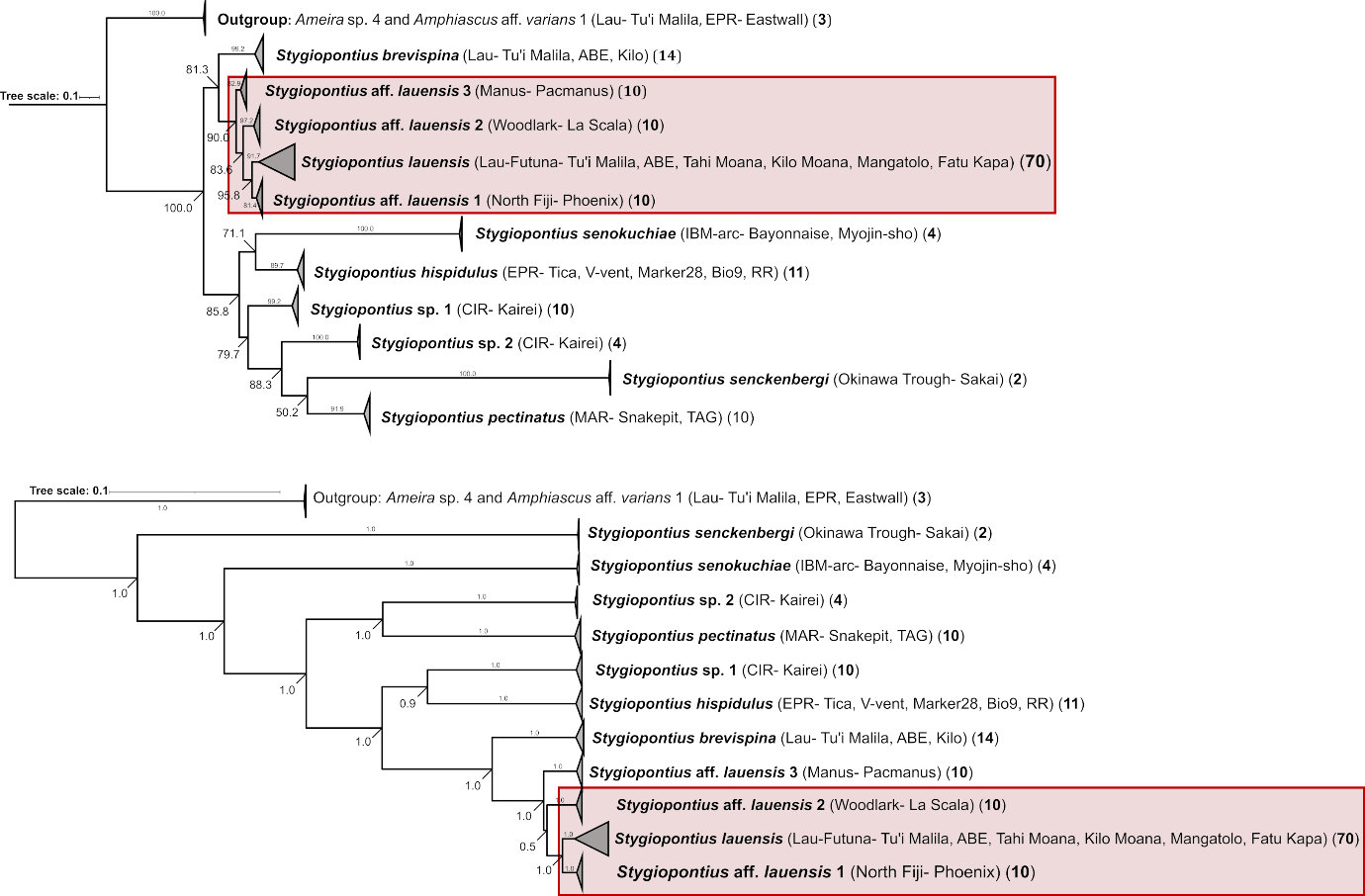


**Fig 2**: Above, the Maximum Likelihood tree found in the main text based on 559 bp of codon position partitioning of the mitochondrial *Cox1* for dirivultid copepods. Tree built with IQ-TREE (node values are ML bootstraps). Below, the Bayesian Posterior Probability (BPP) tree with strict clock (node values are BPP). Tip nodes are proportional to the number of sequences in each clade. Numbers in brackets denote the number of individuals used to build the trees.

**Table 1**: Model parameters as given in the output from Tracer v1.7.2 for the Bayesian tree run for the harpacticoid alignment. For each parameter the mean and the Effective Sample Size (ESS) is given. All parameters are said to have converged when the ESS is above 200.

| **Model parameter** | **Mean** | **ESS** |
| --- | --- | --- |
| posterior | -5241.367 | 617 |
| likelihood | -5554.595 | 2230 |
| prior | 2.258 | 1561 |
| speciescoalescent | 310.97 | 513 |
| birthRate.t:Species | 6.153 | 3161 |
| YuleModel.t:Species | 8.249 | 1369 |
| popMean | 2.80E-03 | 418 |
| TreeHeight.Species | 0.382 | 1284 |
| TreeHeight.t:harps_final_withbathy_1 | 0.42 | 1580 |
| treeLikelihood.harps_final_withbathy_1 | -1283.26 | 1425 |
| treeLikelihood.harps_final_withbathy_2 | -462.648 | 4573 |
| treeLikelihood.harps_final_withbathy_3 | -3808.687 | 2962 |
| treePrior.t:harps_final_withbathy_1 | 176.492 | 1145 |
| clockRate.c:harps_final_withbathy_2 | 9.40E-02 | 3302 |
| clockRate.c:harps_final_withbathy_3 | 10.088 | 2178 |
| rateAC.s:harps_final_withbathy_1 | 0.175 | 5482 |
| rateAG.s:harps_final_withbathy_1 | 0.486 | 1717 |
| rateAT.s:harps_final_withbathy_1 | 0.273 | 2848 |
| rateCG.s:harps_final_withbathy_1 | 0.11 | 5231 |
| rateCT.s:harps_final_withbathy_1 | 4.795 | 1463 |
| rateGT.s:harps_final_withbathy_1 | 0.162 | 3421 |
| substmodel.s:harps_final_withbathy_1 | 1.23E+05 | 7783 |
| rateAC.s:harps_final_withbathy_2 | 0.536 | 7711 |
| rateAG.s:harps_final_withbathy_2 | 1.596 | 6584 |
| rateAT.s:harps_final_withbathy_2 | 0.428 | 7604 |
| rateCG.s:harps_final_withbathy_2 | 1.924 | 6489 |
| rateCT.s:harps_final_withbathy_2 | 1.075 | 7668 |
| rateGT.s:harps_final_withbathy_2 | 0.439 | 8092 |
| substmodel.s:harps_final_withbathy_2 | 1.22E+05 | 8299 |
| rateAC.s:harps_final_withbathy_3 | 0.239 | 6138 |
| rateAG.s:harps_final_withbathy_3 | 2.002 | 1482 |
| rateAT.s:harps_final_withbathy_3 | 0.244 | 3429 |
| rateCG.s:harps_final_withbathy_3 | 0.475 | 3942 |
| rateCT.s:harps_final_withbathy_3 | 2.805 | 1330 |
| rateGT.s:harps_final_withbathy_3 | 0.234 | 4249 |
| substmodel.s:harps_final_withbathy_3 | 1.22E+05 | 7771 |
| BMT_ModelIndicator.s:harps_final_withbathy_1 | 15.12 | 7936 |
| BMT_ModelIndicator.s:harps_final_withbathy_2 | 16.253 | 8206 |
| BMT_ModelIndicator.s:harps_final_withbathy_3 | 12.545 | 6598 |
| BMT_gammaShape.s:harps_final_withbathy_1 | 0.804 | 957 |
| BMT_gammaShape.s:harps_final_withbathy_2 | 0.248 | 1916 |
| BMT_gammaShape.s:harps_final_withbathy_3 | 1.151 | 2492 |
| BMT_ProportionInvariable.s:harps_final_withbathy_1 | 0.277 | 588 |
| BMT_ProportionInvariable.s:harps_final_withbathy_2 | 0.253 | 2270 |
| BMT_ProportionInvariable.s:harps_final_withbathy_3 | 2.30E-02 | 1325 |
| hasGammaRates.s:harps_final_withbathy_1 | 1 | - |
| hasGammaRates.s:harps_final_withbathy_2 | 0.975 | 2456 |
| hasGammaRates.s:harps_final_withbathy_3 | 1 | - |
| hasInvariableSites.s:harps_final_withbathy_1 | 0.777 | 758 |
| hasInvariableSites.s:harps_final_withbathy_2 | 0.683 | 5089 |
| hasInvariableSites.s:harps_final_withbathy_3 | 0.238 | 3904 |
| ActivePropInvariable.s:harps_final_withbathy_1 | 0.262 | 571 |
| ActivePropInvariable.s:harps_final_withbathy_2 | 0.201 | 2228 |
| ActivePropInvariable.s:harps_final_withbathy_3 | 5.44E-03 | 4290 |
| ActiveGammaShape.s:harps_final_withbathy_1 | 0.804 | 957 |
| ActiveGammaShape.s:harps_final_withbathy_2 | 0.212 | 3133 |
| ActiveGammaShape.s:harps_final_withbathy_3 | 1.151 | 2492 |
| hasEqualFreqs.s:harps_final_withbathy_1 | 4.22E-03 | 402 |
| hasEqualFreqs.s:harps_final_withbathy_2 | 0.00E+00 | - |
| hasEqualFreqs.s:harps_final_withbathy_3 | 0.00E+00 | - |
| BMT_frequencies.s:harps_final_withbathy_1.1 | 0.277 | 2119 |
| BMT_frequencies.s:harps_final_withbathy_1.2 | 0.167 | 1991 |
| BMT_frequencies.s:harps_final_withbathy_1.3 | 0.27 | 2336 |
| BMT_frequencies.s:harps_final_withbathy_1.4 | 0.286 | 2103 |
| BMT_frequencies.s:harps_final_withbathy_2.1 | 0.132 | 3509 |
| BMT_frequencies.s:harps_final_withbathy_2.2 | 0.232 | 3644 |
| BMT_frequencies.s:harps_final_withbathy_2.3 | 0.193 | 3511 |
| BMT_frequencies.s:harps_final_withbathy_2.4 | 0.443 | 3052 |
| BMT_frequencies.s:harps_final_withbathy_3.1 | 0.318 | 1362 |
| BMT_frequencies.s:harps_final_withbathy_3.2 | 0.109 | 1706 |
| BMT_frequencies.s:harps_final_withbathy_3.3 | 0.184 | 1573 |
| BMT_frequencies.s:harps_final_withbathy_3.4 | 0.389 | 1284 |


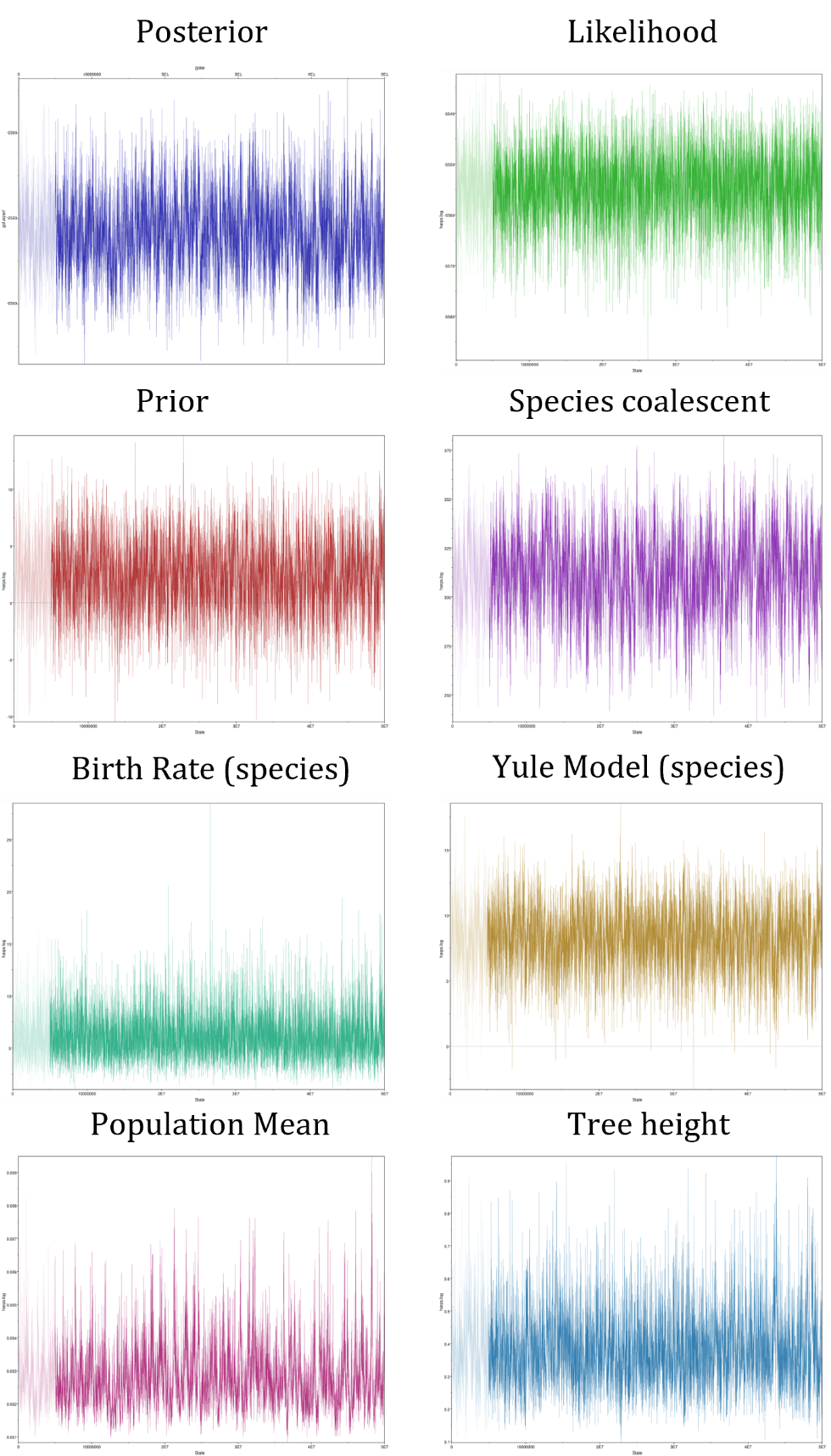


**Fig 3**: Trace files as show in the output of Tracer v1.7.5 for the harpacticoid alignment, for each of the top main model parameters of posterior probability, likelihood, prior, species coalescent, the birth rate for the species tree, the coalescent model for the species tree (Yule), the population mean and the tree height. All parameters show convergence and correspond to values shown in table 2.

**Table 2**: Model parameters as given in the output from Tracer v1.7.2. for the Bayesian tree run for the dirivultid alignment. For each parameter the mean and the Effective Sample Size (ESS) is given. All parameters are said to have converged when the ESS is above 200.

| **Model parameter** | **Mean** | **ESS** |
| --- | --- | --- |
| posterior | -4520.48 | 891 |
| likelihood | -5492.22 | 970 |
| prior | 17.225 | 5855 |
| speciescoalescent | 954.513 | 902 |
| birthRate.t:Species | 10.936 | 7926 |
| YuleModel.t:Species | 22.967 | 2307 |
| popMean | 1.04E-03 | 609 |
| TreeHeight.Species | 0.32 | 1868 |
| TreeHeight.t:truncated_1 | 0.331 | 2086 |
| treeLikelihood.truncated_1 | -1325.32 | 1523 |
| treeLikelihood.truncated_2 | -562.083 | 3273 |
| treeLikelihood.truncated_3 | -3604.81 | 1298 |
| treePrior.t:truncated_1 | 695.917 | 814 |
| clockRate.c:truncated_2 | 0.203 | 12475 |
| clockRate.c:truncated_3 | 11.075 | 1491 |
| rateAC.s:truncated_1 | 0.273 | 7329 |
| rateAG.s:truncated_1 | 0.764 | 1426 |
| rateAT.s:truncated_1 | 0.338 | 905 |
| rateCG.s:truncated_1 | 0.262 | 5817 |
| rateCT.s:truncated_1 | 4.064 | 1141 |
| rateGT.s:truncated_1 | 0.3 | 1559 |
| substmodel.s:truncated_1 | 1.22E+05 | 9080 |
| rateAC.s:truncated_2 | 0.401 | 31269 |
| rateAG.s:truncated_2 | 1.708 | 31449 |
| rateAT.s:truncated_2 | 0.378 | 29053 |
| rateCG.s:truncated_2 | 1.575 | 27470 |
| rateCT.s:truncated_2 | 1.578 | 29570 |
| rateGT.s:truncated_2 | 0.36 | 31114 |
| substmodel.s:truncated_2 | 1.22E+05 | 36001 |
| rateAC.s:truncated_3 | 0.192 | 7182 |
| rateAG.s:truncated_3 | 1.827 | 6986 |
| rateAT.s:truncated_3 | 0.165 | 1456 |
| rateCG.s:truncated_3 | 0.184 | 6089 |
| rateCT.s:truncated_3 | 3.477 | 3015 |
| rateGT.s:truncated_3 | 0.156 | 2466 |
| substmodel.s:truncated_3 | 1.22E+05 | 32611 |
| BMT_ModelIndicator.s:truncated_1 | 12.801 | 11999 |
| BMT_ModelIndicator.s:truncated_2 | 14.867 | 36001 |
| BMT_ModelIndicator.s:truncated_3 | 11.126 | 32747 |
| BMT_gammaShape.s:truncated_1 | 0.672 | 7162 |
| BMT_gammaShape.s:truncated_2 | 0.328 | 6639 |
| BMT_gammaShape.s:truncated_3 | 1.846 | 3057 |
| BMT_ProportionInvariable.s:truncated_1 | 0.182 | 6594 |
| BMT_ProportionInvariable.s:truncated_2 | 0.251 | 6914 |
| BMT_ProportionInvariable.s:truncated_3 | 1.26E-02 | 3148 |
| hasGammaRates.s:truncated_1 | 1 | 18002 |
| hasGammaRates.s:truncated_2 | 0.986 | 10233 |
| hasGammaRates.s:truncated_3 | 1 | - |
| hasInvariableSites.s:truncated_1 | 0.714 | 10152 |
| hasInvariableSites.s:truncated_2 | 0.695 | 15276 |
| hasInvariableSites.s:truncated_3 | 8.18E-02 | 27891 |
| ActivePropInvariable.s:truncated_1 | 0.158 | 6396 |
| ActivePropInvariable.s:truncated_2 | 0.21 | 6912 |
| ActivePropInvariable.s:truncated_3 | 1.02E-03 | 29285 |
| ActiveGammaShape.s:truncated_1 | 0.672 | 7181 |
| ActiveGammaShape.s:truncated_2 | 0.308 | 8642 |
| ActiveGammaShape.s:truncated_3 | 1.846 | 3057 |
| hasEqualFreqs.s:truncated_1 | 6.99E-02 | 351 |
| hasEqualFreqs.s:truncated_2 | 0.00E+00 | - |
| hasEqualFreqs.s:truncated_3 | 0.00E+00 | - |
| BMT_frequencies.s:truncated_1.1 | 0.309 | 3491 |
| BMT_frequencies.s:truncated_1.2 | 0.168 | 1702 |
| BMT_frequencies.s:truncated_1.3 | 0.251 | 7627 |
| BMT_frequencies.s:truncated_1.4 | 0.272 | 8272 |
| BMT_frequencies.s:truncated_2.1 | 0.128 | 14635 |
| BMT_frequencies.s:truncated_2.2 | 0.258 | 13658 |
| BMT_frequencies.s:truncated_2.3 | 0.176 | 13075 |
| BMT_frequencies.s:truncated_2.4 | 0.438 | 12491 |
| BMT_frequencies.s:truncated_3.1 | 0.323 | 5986 |
| BMT_frequencies.s:truncated_3.2 | 0.118 | 6408 |
| BMT_frequencies.s:truncated_3.3 | 0.153 | 6488 |
| BMT_frequencies.s:truncated_3.4 | 0.406 | 5753 |


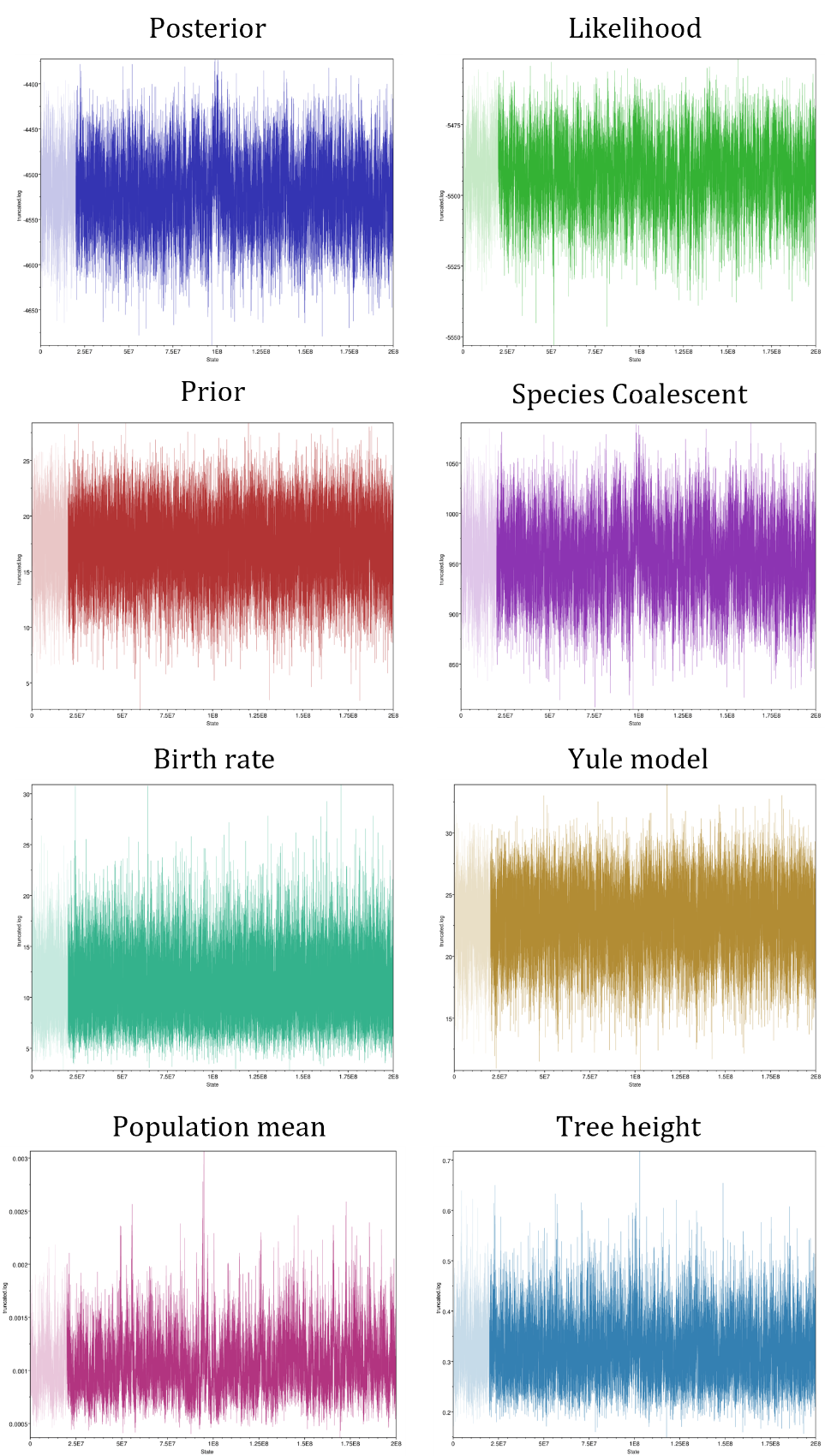


**Fig 4**: Trace files as show in the output of Tracer v1.7.5 for the dirivultid alignment, for each of the top main model parameters of posterior probability, likelihood, prior, species coalescent, the birth rate for the species tree, the coalescent model for the species tree (Yule), the population mean and the tree height. All parameters show convergence and correspond to values shown in table 2.
