# Supplementary material for: Highly structured populations of deep-sea copepods associated with hydrothermal vents across the Southwest Pacific, despite contrasting life history traits
Source: PLoS One. 2023 Nov 6;18(11):e0292525. doi: 10.1371/journal.pone.0292525 (PMC10627453; doi:10.1371/journal.pone.0292525)
Supplement: Attachment — Submitted filename: paper.pdf [file pone.0292525.s007.pdf]

Abstrat:

Line 41: I find the selected sites that do not have any hydrothermal effect to be biased, as there are 22 sites with an effect and only 3 without it.

Introduction:

Line 77: I don't understand what is meant by "50% biodiversity" in relation to what.

Line 88: It discusses the abundance of copepods in hydrothermal vents, but there is no citation to support it. However, later on, some works are mentioned. I suggest adding a citation to this line.

Line 99: The specific traits are not specified. Which traits are being referred to? It might be better to reorganize this paragraph, starting with the general aspects and then addressing the specific ones.

Materials and Methods:

Table 2 is misaligned, please adjust it. Additionally, the sampling equipment used in each zone could also be added.

After Table 2, the paper is not enumerated . I suggest adding the corresponding enumeration.

In Table 2, it would be helpful to include the sampling method used instead of mentioning it only in the text, or at least briefly mention it.

The samples were not preserved in cold storage. Does this help prevent degradation of their genetic material?

I would change the title "Species identification" to "Species identification using classical taxonomy."

In the Materials and Methods section, it is not recommended to present the results, but species found are mentioned here. I recommend moving this to the Results section. In this section, mention the taxonomic references used and cite them.

Chelex has a standard protocol, what is it? Rather than a "standard protocol," it is the protocol provided by the manufacturer.

Both primer pairs had the same annealing temperature. In what cases was each one used? What was it dependent on?

Data Analysis:

It would be convenient to create a table that includes the accession codes of all the sequences used previously and the new ones, along with their taxonomic assignment and the reference of the work in which they were published.

What type of BLAST was used? Nucleotide or amino acid?

The delimitation within a species and to determine if a species is cryptic is between 2% and 5%. You mention 20%, which could correspond to differences at the level of different families. This point is very important.

Construction of the phylogenetic tree:

Please review references 61 and 65, which do not discuss species delimitation. In general, for delimitation, a tree is input into PTP and the alignment into BEAST to generate an ultrametric tree. In the former, patterns of substitution at the nucleotide level are observed, not at the amino acid level. The latter, BEAST, offers around 4 different models that are the most common. Typically, consensus is used to build trees, without considering the reading frame. However, efforts are made to equalize the length to avoid such problems. I feel that using different models and trees generates more problems when it comes to interpretation.

In the Bayesian and Maximum Likelihood trees, not all input parameters are indicated, such as the number of iterations, the number of chains, the number of burn-in steps, and the sampling frequency. I believe it is of utmost importance to specify this in the methods.

The external groups used should be indicated by their accession code, and I don't find them explicitly mentioned. It would be convenient to explicitly state them in the text.

In the species delimitation analyses, only the methods used are mentioned, but it is not indicated which trees were used or the input values. I believe, once again, that it is important to include this in the Materials and Methods section.

Molecular and demographic variance analysis:

The input parameters and the alignment used are not indicated again, given that different models of evolution were calculated.

Tree calibration: Where was it extracted from? What bibliographic source supports this? What program was used? What were the other input parameters? Which alignment was used?

In citation 28, the number does not allow identification of whom it refers to.

Results:

Dirivultidae:

It cannot be solely claimed through phylogeny that a species complex exists. If you wish to assert that, I would first cite the results, such as bTPP.

Is the indicated FST value significant? Was a probability calculated for the FST?

The difference between the Bayesian and Maximum Likelihood trees is due to them being different tree inference methods.

In Figure 2, I cannot distinguish which is the Bayesian probability and which is the maximum likelihood probability.

Ameiridae:

In Figure 3, when "BEAST three" is mentioned, does it refer to a Bayesian tree? Please specify.

Miraciidae:

The FST value alone does not say much. It is important to indicate the significance, which is not mentioned here.

Molecular diversity:

Some probability values are not indicated in the FST and AMOVA in the text.

Table 3 is not fully displayed.

Table 4 is not fully displayed.

Discussion:

When it is mentioned that "The Da values are approximately twice as high between S. lauensis and S. aff. lauensis 1 as between S. aff. lauensis 2 and 3, suggesting that divergence took twice as long for the latter two allopatric forms under a constant molecular clock [81]," it is challenging to reach that conclusion if different molecular clocks were not tested or if literature supporting the appropriate molecular clock usage within the genus is not cited.

^{14}C is not indicative of the food source but rather of time, although ^{13}C could provide information about the food source.

It would be important to mention the issue of working with the selected marker for studying structuring and note that there are other analyses with better resolution, such as the use of microsatellites or mitochondrial genome sequencing.

I did not see any comparison of the obtained sequences with sequences from shallower species in the analysis.
