# Supplementary material for: Highly structured populations of deep-sea copepods associated with hydrothermal vents across the Southwest Pacific, despite contrasting life history traits
Source: PLoS One. 2023 Nov 6;18(11):e0292525. doi: 10.1371/journal.pone.0292525 (PMC10627453; doi:10.1371/journal.pone.0292525)
Supplement: Attachment — Submitted filename: Lorenzo et al review.docx [file pone.0292525.s005.docx]

**Review of Lorenzo et al. PlosOne**

Lorenzo et al. did a very thorough study of deep-sea copepods population structure and demography across the Southwest Pacific using the mitochondrial cytochrome oxidase I gene. After identifying specimens morphologically, they performed a phylogenetic analysis and discover cryptic species in Stygiopontius lauensis. They then further their sampling in the back-arc basins for phylogeographical and demographical analyses. They found population structure within species and evidence of population expansion hypothesized being linked to an increase of hydrothermal vent activities after the Last Glacial Maximum.

This paper is very long and could have been split into multiple papers (either per copepod family, or phylogeny versus phylogeography). However, because of the comparative aspect of the study, I understand the authors’ desire to incorporate the complete study into a single comprehensive paper. The paper would nevertheless benefit from some reorganizations that would make it an easier flow, but these re-organizations should be easy to achieve.

I recommend accepted the paper after minor revisions. Below are my comments and edits.

**Introduction**

Line 69 – “~~and~~ as well as”

Line 74 – add references to the papers

Line 160-163- should refer to Fig. 1 to help the reader

**Materials and Methods**

Line 174 – “kapa” to “Kapa”

Line 176 – I would refer to Fig 1 and table 2 together here, so readers can find site names more easily.

Line 177 – “along the EPR” is a bit misleading since all sampling sites are from 9N. Replace “along the East Pacific Rise” by “from the East Pacific Rise”

Line 179 – Spell out “Izu–Bonin–Mariana (IBM)”

Unfortunately, the line numbers were not provided after this point

Page 14 – Missing closing parenthesis “refer to [18, 19, 57, 58]” to “refer to [18, 19, 57, 58])”

Page 15 – Explain in the methods why only the females were dissected. It’s not clear at this point why the dissection is needed for the study. Based on the result section, I understand the female have specific “characters that discriminate” them from other species. It would help knowing this when reading the methods.

Page 15 - “pictures taken with the Leica Application suite”, however pictures were not provided with the paper. Either provide the picture (supplementary material) or remove the end of the sentence as it is not needed for the study.

Page 15 – please add PCR mix conditions, especially since you are using 2 sets of primers. Or indicate you followed the PCR mix conditions from [59] if that is the case.

Page 16 – which primer pairs were used to sequence?

Page 16 – The way the cleaning of sequences is written is confusing. If ambiguities were resolved appropriately using the chromatograms, then there should not be any gaps (insertion/deletion), nor frame shift. If, as stated, some sequences had to be removed because of frame shift and stop codon found after cleaning the sequences makes me wonder if the all the used cleaned sequences were of good enough qualities or if some mutation may be the result of sequences that have not been sufficiently verified using the chromatograms. If chromatograms were not used in the initial cleaning, then authors should verify them to ensure all sequences used in further analysis are indeed unambiguous.

Page 16 – “blasted against the NCBI database […] allowed to differ by 20%” Which blast was used? What parameter was used with regards to the 20%? The e-value?

Page 18 – “Further, ….. between two models” incomplete sentence

Page 19 – Need to make it clear analyses of diversity and demography are being done among populations of the same species or species complex

Page 19 – “DnaSP was used to first define populations” 🡪 based on vent sites? Clarify

Page 20 – “To further investigate (…) Cox1 gene. Extended” replace the dot by a coma

Page 20 – “[82] and were constructed” missing information or remove “and”

**Results**

**Comments**:

While the order of the method section should remain the same, I found myself lost in the result section. The result section would be better re-organized per family as suggested below. It would make for an easier flow to fully understand an entire family before moving onto the next family. For example, while reading the molecular diversity and looking at haplotype networks in the Dirivultidae, I found myself wanting to know the demography of that family, which doesn’t come until much later in the paper.

As is, the results are difficult to follow, especially because figures and tables are sometime out of order. Figures/tables, including supplementary material, should be renumbered to follow the order they are cited in the text.

The authors need to provide the accession numbers of the sequences.

*Suggested order for the result section:*

1. *Dirivultidae*
   1.1. *Morphological identification of the S. lauensis species complex*
   1.2. *Phylogenetic reconstruction and species delimitation*
   1.3. *Molecular diversity (including haplotype networks)*
   1.4. *Demography*
2. *Ameiridae*
   2.1. *Phylogenetic reconstruction and species delimitation*
   2.2. *Molecular diversity (including haplotype networks)*
   2.3. *Demography*
3. *Miraciidae*
   3.1. *Phylogenetic reconstruction and species delimitation*
   3.2. *Molecular diversity (including haplotype networks)*
   3.3. *Demography*

**Edits**:

Page 20 – Results: italicize Stygiopontius lauensis

Page 21 – Change to: The phylogeny of *S. lauensis* reconstructed using X bp-sequences revealed…

Page 21 and through – Stay consistent in the entire manuscript “Table” or “table”

Page 22 – After the end of the Dirivultidae paragraph, please describe the network shown in Fig 4 and 5.

Page 22 - A sp.4 and A sp5 are inversed in the text. A sp. 5 on the figure is the one from East Wall and A sp.4 from ABE.

Page 22 – The figures numbering if off. Fig 6, (not Fig 5) has Ameira. Also, there is no mention in the text of Fig 4 and 5 before

Page 24: italicize Stygiopontius

Page 24 – Period after (Fig 4c and d).

Page 27 – 6 sequences for *A.* aff *varians* 1 is small to conclude there is “no structure within the species”. Also, both sites are from 9°N. You may detect structure within the species if you had samples from the entire EPR. I would rephrase that you did not detect structure, but higher sampling size and broader sampling area are needed.

**Discussion**

**Comment:**

Page 32 – “This is in contrast to *Stygiopontius* from MORs, …. population structure occurring only in BABs”, and page 33 “only in the species belonging to BABs”, and page 43-44 “Results point to … than on MORs for vent-endemic copepods”

The authors made strong statements of population structure being much stronger in BAB than in MORs. I wouldn’t make such strong statements as it may just be due to differences in sampling scales in the study. There is a very different geographical scale between the sampling area in the BABs (>17° of latitude from Manus to Lau) versus the MORs. In *Stygiopontius hispidulus*, the authors only had samples from 9°N (EPR) and a single sequence from GC. Had they sampled along the entire EPR, they may have found a greater diversity and greater population structure in *Stygiopontius hispidulus* they were not able to capture with their current samples. This is especially the case as 9°N and GC are both north of the equator, and south EPR is a different biogeographical region. Similarly on MAR, TAG and Snake Pit are only 3° of latitude apart.

**Edits:**

Page 33 – “this could to asymmetric gene flow” 🡪 missing verb

Page 37 – Add “(LGM)” after Last Glacial Maximum

Through the paper: be consistent in using either UK English or US English. For example, specialization/specialisation (page 38), among/amongst,

Page 38 – “Dirivultids are vent-specialists … *Benthoxinus scupilifer*”. This paragraph feels untight to the study. A sentence is needed to explain why being vent specialists, and the specialization gradient is important in the context of this genetic study and the discussed effective population size (or would that paragraph be better moved after discussing the population structure?).

Page 41 “in in”

**Tables and Figures**

Figures/tables, including supplementary material, should be renumbered to follow the order they are cited in the text.

**Tables 2, 3 and 4**

Because of formatting, I was not able to see the entire tables. Tables was cut-off.

**Fig 1.**

Add “Futuna Volcanic Arc” on the 1.b map.

It would help readers who are unfamiliar with deep-sea sites if authors could link the sampling sites on the map to the ones listed in Table 2. For example, authors could add a superscript number by each vent field and add that superscript number next to the vent field in Table 2.

North Sea^1^, Hawaii^2^, etc

**Fig. 4, 5 and 6**

Re-order the sites in the colored legends based on geographical location (north to south). Keep the same order in the figure legend description.

**Fig 7**

Legend: There is no “red dotted line”. You have yellow (constant) and blue/green (expansion).

**Fig 8**

Legend: “dashed black line” and “straight black line” do not correspond to the figure 🡪 change to “black line” and “shaded grey area”.
