# Supplementary material for: Highly structured populations of deep-sea copepods associated with hydrothermal vents across the Southwest Pacific, despite contrasting life history traits
Source: PLoS One. 2023 Nov 6;18(11):e0292525. doi: 10.1371/journal.pone.0292525 (PMC10627453; doi:10.1371/journal.pone.0292525)
Supplement: Attachment — Submitted filename: Review1.docx [file pone.0292525.s006.docx]

This manuscript is a study of phylogenetic structure and life history traits of hydrothermal vent-associated copepods, one of the most abundant and diverse members of vent communities which is nonetheless very poorly known. It includes inter- and intraspecific phylogenetic structure based on CO1 data from 14 copepod species (plus 4 newly identified cryptic species) spanning 4 copepod families collecting comprising 708 specimens from 25 sites and corresponding trait data for each family. It is a valuable contribution to the scientific literature both in terms of copepod phylogeny and population structure and is also of broader interest to deep sea biology and hydrothermal vent communities.

I have number of minor suggestions:

1. I assume the authors plan to do this, but all new sequences should be uploaded to NCBI.
2. A table should be provided in the supplemental information that includes the taxonomic information, collection metadata, and NCBI numbers for all the new sequences.
3. I did not see the figure captions for the figures in the main text. These should be checked carefully. Especially for the phylogenetic tree figures, it is critical that the tree is reported in the captions (i.e., which tree is displayed, the highest likelihood tree from ML analysis of the consensus tree from BI analysis) and how the support values are given (support values appear to be BS/PP in some figures or just PP in others).
4. Throughout the manuscript the genus name for *Ameira* and *Amphiascus* should be written out in full, not abbreviated to *A*. in sections of the text where both taxa are referred to since it can be difficult for the reader to follow which genus is being discussed. (e.g., page 14)
5. When used at the start of a sentence, the full genus name should be written out, not abbreviated (e.g., *A*. sp. 5 on page 26 and A. aff. varians 1 on page 27).
6. When a shortened taxon name is used (e.g., dirivultid or harpacticoid) it should not be capitalized unless it is at the start of a sentence (e.g., page 32).
7. Standardize usage of “cox1” vs “Cox1” throughout the manuscript. I would suggest simply “CO1” although any of the above are acceptable, just be consistent.
8. Remove the following lines form the Phylogenetic Reconstruction section of Methods since this is more of an explanation of common phylogenetic knowledge and not a detail of the methods.

“Codon-usage bias can result in different mutation rates for different coding regions of a gene and therefore, different evolutionary histories for each coding region which, if not modelled correctly can lead to erroneous phylogenies and interpretation of species delimitation methods [61-65]. Therefore…”

Some details are missing from the Phylogenetic Reconstruction Methods section and it should be expanded to include:

9. Details of ML analysis: what tree is shown presented? The maximum likelihood tree or the majority rule consensus (the highest likelihood tree should be presented).
10. Details of the BI analysis (a) number of chains (b) number of generations (c) frequency of sampling (d) amount of burn-in generations (e) how convergence between chains was assessed (visually with tracer or statistically?).
11. Support value information. How many BS replicates in ML analysis?
12. Species delimitation methods: For BPTP wow many MCMC generations were completed (should be 100,000 at the minimum) and what was the sampling frequency and burn-in?
13. I recommended rewording the following sentence on page 21 to switch the word order from “The other Stygiopontius species had been morphologically previously identified by…” to “The other Stygiopontius species had been morphologically identified previously by…”
14. In some cases, the phrase “exist only at depth” (e.g., page 33, 35) is used. Specify what you mean by “at depth”. At depths over 1,000m for example?
15. Page 38 put “e.g., S. quadrispinosus” in parentheses.
16. Page 43, Conclusions. To emphaise which 2 groups of copepods you are referring to, I would reorganize how the taxa are mentioned from:

“Two main groups of copepods, the Dirivultidae (Siphonostomatoida) and the Ameiridae, Miraciidae, and Bathylaophontidae (Harpacticoida)…”

to:

“Two main groups of copepods, the Siphonostomatoida (Dirivultidae) and Harpacticoida (Ameiridae, Miraciidae, and Bathylaophontidae)…”

17. Page 44. Consider rewording the following sentence, it sounds a bit awkward: “…to accurately determine population genetic parameters of structure and demography”
18. Page 44. “Can help evaluating” should be changed to “Can help evaluate” or “Can help with evaluating”.
